# Supplementary material for: Chromothripsis during telomere crisis is independent of NHEJ, and consistent with a replicative origin
Source: Genome Res. 2019 May;29(5):737–49. doi: 10.1101/gr.240705.118 (PMC6499312; doi:10.1101/gr.240705.118)
Supplement: Supplemental Material [file supp_gr.240705.118_Supplemental_file_1.zip › contigs/annotated_contigs/DB79/contig.2.DB79_length_634_mean_cov_12.5394321767.docx]

**DB79_length_634_mean_cov_12.5394321767**

```
CTGTGCTATAAGAAATGTTAAAAGGAGTCCTTCAGACAGGTCAATTATATAAGTCACAAACTTGTGTCTACATAGAAAATGAAAGAGAC
 >chr13:83301915-83302249 - E=3e-189 p=4e-02
TCAGAGAAAGAATAAATGAAGATAAGGTAAAATCTTTTTCTCAATGTTAAATTATCTAAAGAATGACCGCGTAAAACAATAATAACAGT

AACAATGCACTGAGTAATTATAGCATATGAGTAAGTGAAATAAATGACAACAATATCAAAGGGTCTGGAGGAAGAAATTGGAGGAAAAA

AAACTATTGTATAAGGTTATAATCAAAGACAACAACACACCCACAGACACATACATACACACACAT|A|TGAAACGTATGAAAGCAGAA
                                                                   >chr13:82788135-8278
AAAGGGGAAAAGCTATGTGACAATGGCCTGAGCAATGATTTTTTCTGGATTTGACCCTGAAAGCACAGGCAACAAAAGCAAAAATAGGT
8436 - E=2e-169
AAATGGGATTATACCAGACTAAAAAATCTTCTGAACAGCAAATGAAATAGTTAACAGTGTGAACTAACAACCTACAGACTGGGAGAAAA

TATTTGCAAGCCATATATCTGATAGGGAATTAATATCCAAAATATATAAGTAGTTTACACAACTCAATAACAAACCAAACCAAAGAAAC

AAACAAGTAAAAG
```
